# Hydroxychloroquine as a Chemoprophylactic Agent for COVID-19: A Clinico-Pharmacological Review

**DOI:** 10.3389/fphar.2020.593099

**Published:** 2020-12-17

**Authors:** Mudit Agarwal, Piyush Ranjan, Upendra Baitha, Ankit Mittal

**Affiliations:** ^1^ MBBS, All India Institute of Medical Sciences, New Delhi, India; ^2^ Department of Medicine, All India Institute of Medical Sciences, New Delhi, India

**Keywords:** severe acute respiratory syndrome coronavirus 2, hydroxychloroquine, coronavirus disease 19, chemoprophylaxis, coronavirus

## Abstract

Hydroxychloroquine has gained much attention as one of the candidate drugs that can be repurposed as a prophylactic agent against SARS-CoV-2, the agent responsible for the COVID-19 pandemic. Due to high transmissibility and presence of asymptomatic carriers and presymptomatic transmission, there is need for a chemoprophylactic agent to protect the high-risk population. In this review, we dissect the currently available evidence on hydroxychloroquine prophylaxis from a clinical and pharmacological point of view. *In vitro* studies on Vero cells show that hydroxychloroquine effectively inhibits SARS-CoV-2 by affecting viral entry and viral transport via endolysosomes. However, this efficacy has failed to replicate in *in vivo* animal models as well as in most clinical observational studies and clinical trials assessing pre-exposure prophylaxis and postexposure prophylaxis in healthcare workers. An analysis of the pharmacology of HCQ in COVID-19 reveals certain possible reasons for this failure—a *pharmacokinetic* failure due to failure to achieve adequate drug concentration at the target site and attenuation of its inhibitory effect due to the presence of TMPRSS2 in airway epithelial cells. Currently, many clinical trials on HCQ prophylaxis in HCW are ongoing; these factors should be taken into account. Using higher doses of HCQ for prophylaxis is likely to be associated with increased safety concerns; thus, it may be worthwhile to focus on other possible interventions.

## Introduction

COVID-19, the disease caused by the coronavirus SARS-CoV-2, continues to be an immense challenge for the scientific community throughout the world. The number of cases and deaths has been on the rise, but currently, there are only a few therapeutic and no chemoprophylactic interventions in our arsenal to combat the virus.

Due to the constraints of time, there has been much focus on the strategy of “drug repurposing/repositioning,” defined as identifying new uses of approved drugs that are outside the scope of their original medical indication (Ashburn and Thor, 2004). The 4-aminoquinoline hydroxychloroquine (HCQ) and its congener chloroquine (CQ) have been repurposed for COVID-19 due to their proposed antiviral properties (Savarino et al., 2003). Reports of preclinical evidence of efficacy led to HCQ receiving unprecedented attention by the scientific community as well as by the lay public and media. The political attention and the controversies surrounding this drug have further fueled a debate in the scientific community over its potential as a chemoprophylactic agent against SARS-CoV-2 and for treatment of COVID-19.

In this review, we aim to discuss the potential role of HCQ as a chemoprophylactic agent for COVID-19. We discuss why HCQ is a good candidate for a chemoprophylactic agent, followed by a dissection of the currently available evidence. The next section emphasizes the current caveats in knowledge and the complexities associated with HCQ prophylaxis with regard to its dosing and pharmacokinetic properties. Finally, we conclude with an overall assessment of the current evidence and recommendations for the future.

## Need for Prophylaxis in COVID-19

Chemoprophylaxis has been used in many diseases to protect high-risk groups from severe disease, such as malaria prophylaxis for patients with sickle cell disease (Oniyangi and Omari, 2019) and antiviral prophylaxis against influenza for immunosuppressed children and adults (Uyeki et al., 2019) and as a preventive measure against mass outbreaks, for example, mass prophylaxis against meningococcal infections (McNamara et al., 2018). For effective chemoprophylaxis, the drug should have activity against the infective agent and achieve tissue specific concentrations. In addition, adverse effects should be minimal to ensure acceptability. Further, the drug should be easily available and inexpensive.

Certain characteristics of SARS-CoV-2 and COVID-19 have fueled the ongoing pandemic, particularly its high transmissibility and low overall case fatality rates. Estimates for the basic reproduction number (R_0_) of SARS-CoV-2 have ranged from 2 to 5.5 (Li et al., 2020; Read et al., 2020; Shen et al., 2020; Wu et al., 2020), higher than that of SARS-CoV (R_0_ = 1.7–1.9) and MERS-CoV (R_0_ = 0.7) (Petrosillo et al., 2020). More than 80% of COVID-19 cases report only mild symptoms (Wu and McGoogan, 2020). In contrast to SARS, patients with COVID-19 demonstrate high viral loads with active viral replication in the upper respiratory tract (Wölfel et al., 2020), with a peak of viral load occurring at the time of presentation (To et al., 2020). Moreover, recent evidence has suggested that presence of pre-symptomatic transmission and asymptomatic carriers may be common in COVID-19 (Arons et al., 2020; Chau et al., 2020). These characteristics render case-based detection less effective and add to the enigma of controlling the rampant spread of this pandemic. While nonpharmaceutical interventions like case-based isolation, contact tracing, closure of public places, and lockdowns have been able to reduce the spread to an extent (Davies et al., 2020; Patel et al., 2020), proper implementation of these measures is seldom possible for prolonged periods due to the socioeconomic fallout. Thus, in the absence of an effective vaccine in the near future, a chemoprophylactic agent can greatly help in mitigating the impact of COVID-19.

Such an agent should be targeted toward protecting the most susceptible and vulnerable groups within the population. Severe illness and hospitalization due to COVID-19 is known to be associated with older age and presence of comorbidities like diabetes mellitus, hypertension, cardiovascular disease, chronic lung disease, malignancy, and obesity (Cummings et al., 2020; Petrilli et al., 2020; Richardson et al., 2020). The incidence of noncommunicable diseases is increasing worldwide; all-age prevalence of diabetes is projected to rise to 4.4% by 2030, with nearly 366 million cases (Wild et al., 2004). Around one-fourth of the Indian population suffers from hypertension (Gupta et al., 2019), and the prevalence of diabetes and chronic obstructive pulmonary disease is 20.4 and 4.2%, respectively (Salvi et al., 2018; Tandon et al., 2018).Thus, a significant proportion of the population is at risk of severe COVID-19 infection. Close contacts of patients confirmed to have COVID-19 are also at significant risk of contracting it. Another susceptible group that needs to be protected is the healthcare workers (HCW). During the SARS epidemic, most outbreaks occurred in the healthcare setting (Yu et al., 2007). Reports from Italy have shown that as many as 20% HCW taking care of COVID-19 patients were infected (Lancet, 2020). HCW are at increased risk due to prolonged exposure to a large number of infected patients; this risk is compounded if they are involved in performing aerosol-generating procedures like endotracheal intubation or if they are wearing inadequate personal protective equipment (PPE). It is of paramount importance to protect frontline workers in order to prevent overburdening of a country's healthcare system. An effective chemoprophylactic agent is therefore the need of the hour.

In view of this overwhelming need of a chemoprophylactic agent, the exceptional circumstances created by the pandemic, and preliminary evidence of efficacy of HCQ, the COVID-19 National Task Force of India issued a recommendation for empiric use of HCQ as prophylaxis for all HCW, other frontline workers involved in COVID-19 activities, and asymptomatic household contacts of laboratory-confirmed cases (National Task Force for COVID-19, 2020). The dosage recommended was a loading dose of 400 mg twice a day on day 1, followed by 400 mg once weekly. There are no official guidelines for hydroxychloroquine prophylaxis in other countries, although off-label use of hydroxychloroquine has been reported in Africa, France, and the United States.

## Mechanism of Action of Hydroxychloroquine and Preclinical Evidence

SARS-CoV-2 enters host cells through binding of the S1 subunit of its spike (S) protein with the ACE2 receptor on the host cell (Hoffmann et al., 2020b). ACE2 binding and subsequent viral fusion requires *priming* of the S protein via proteolytic cleavage by host enzymes. Similar to SARS, S protein priming for entry into human lung epithelial cells of SARS-CoV-2 is enabled by TMPRSS2, a transmembrane serine protease (Hoffmann et al., 2020b). S protein priming can also occur via secondary pathways, such as via endolysosomal cysteine proteases cathepsins B and L; while this path is not of prime importance for viral transmission and respiratory infection. As the human airway epithelium lacks sufficient endolysosomal proteases, it is thought to contribute to invasion of extrapulmonary tissues (Park et al., 2016; Hoffmann et al., 2020b). Further, SARS-CoV-2 S protein can be *preactivated* by furin during packaging of viral particles; this has a cumulative effect on subsequent S protein activation by TMPRSS2 (Hoffmann et al., 2020a; Shang et al., 2020).

**FIGURE 1:**
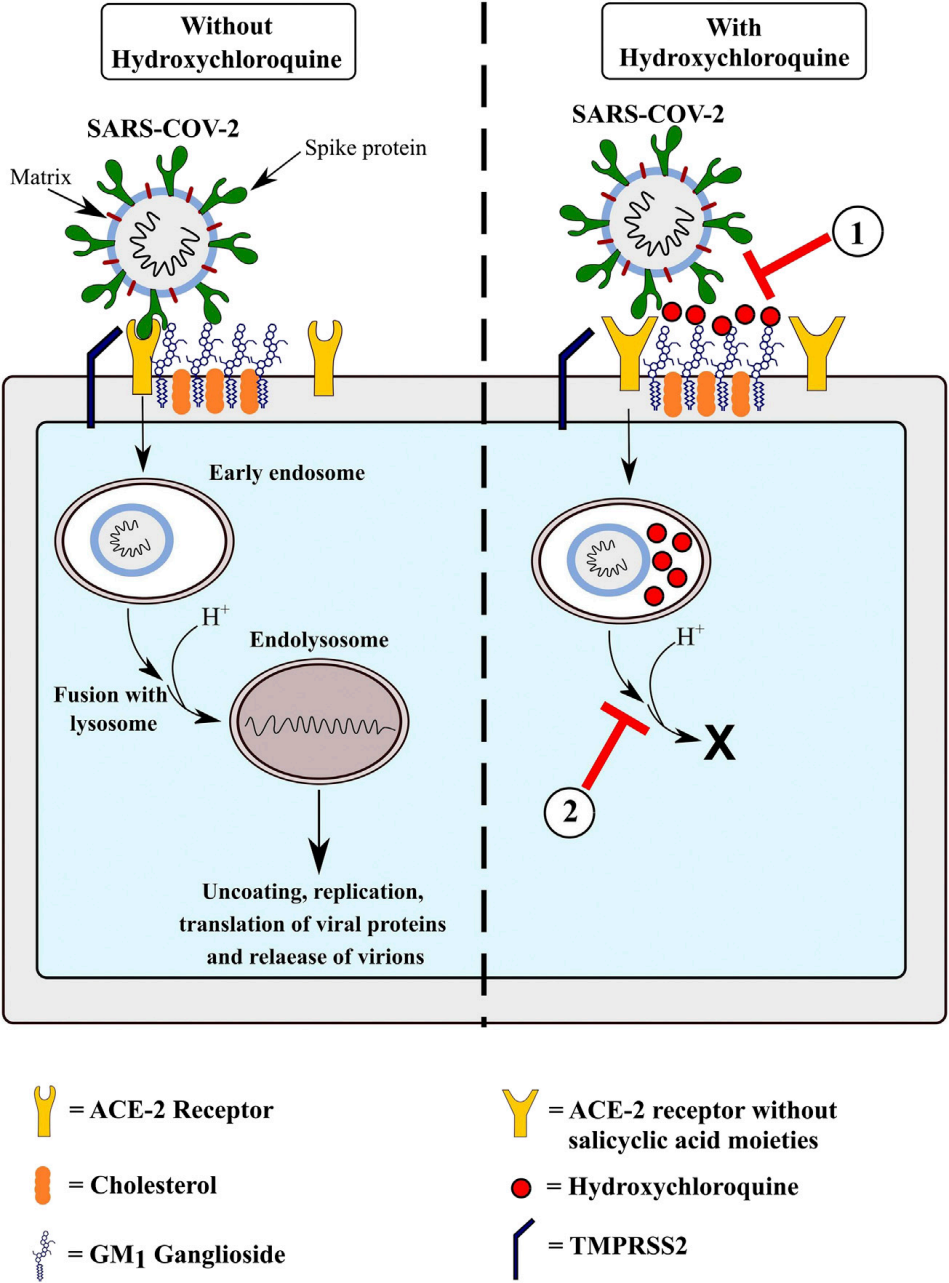
Proposed mechanisms of action of hydroxychloroquine in preventing COVID-19 infection. 1) Hydroxychloroquine blocks entry of SARS-CoV-2 by binding to GM_1_ gangliosides present on the cell membrane, preventing the interaction of the N-terminal domain of the virus’ Spike protein with them. In addition, by inhibiting synthesis of sialic acid moieties on the ACE-2 receptor, it reduces binding of the virus to its target receptor. 2) Hydroxychloroquine is concentrated inside the endosomes and lysosomes in the cell. Due to their basic nature, they decrease the pH inside the endosomes, thus preventing maturation of early endosomes into endolysosomes and preventing the activity of cathepsins B and L.

HCQ can inhibit SARS-CoV-2 by impacting viral entry and postentry steps (Figure 1). By inhibiting glycosylation, it affects synthesis of sialic acid moieties of ACE2 and the terminal glycosylation of the S protein, thereby reducing the interaction between ACE2 and the S protein (Savarino et al., 2006; Liu et al., 2020). *In silico* analyses have revealed that similar to other coronaviruses, the N-terminal of the S protein consists of a ganglioside-binding domain (Fantini et al., 2020b). This domain binds to sialic acid residues linked to GM1 ganglioside cell surface receptors, facilitating binding at ACE-2. HCQ binds to these gangliosides with a high affinity, thus further inhibiting SARS-CoV-2 entry (Fantini et al., 2020b; Fantini et al., 2020a). Being a weak base, HCQ concentrates in the acidic lysosomes and endosomes. By increasing endosomal pH, it inhibits endosomal maturation and fusion of viral and endolysosomal membranes (Derendorf, 2020; Liu et al., 2020). Further, by the same mechanism, it decreases activity of endolysosomal cathepsins. The immunomodulatory action of HCQ is also believed to play a role- HCQ inhibits MHC class II expression, production of pro-inflammatory cytokines like IL-1 and TNF-alpha and inhibits TLR signaling pathways (Schrezenmeier and Dörner, 2020). This anti-inflammatory action can counter the cytokine storm responsible for severe COVID-19 and reduce severity of infection, although this remains but a hypothesis.

*In vitro* studies have demonstrated that HCQ effectively inhibits SARS-CoV-2. Pretreatment of Vero cells with HCQ inhibits SARS-CoV-2 replication with a half-maximal effective concentration (EC_50_) in the range of 4.51–5.85 µM (Liu et al., 2020; Yao et al., 2020). However, this result was not replicated when a model of reconstituted human airway epithelial cells was used; HCQ did not affect apical viral titers and could not protect epithelial integrity (Maisonnasse et al., 2020). This contradiction may be explained by the role of TMPRSS2, which is not expressed in Vero cells. It has been seen that expression of TMPRSS2 attenuates the inhibition of SARS-CoV-2 by HCQ, possibly by bypassing the cathepsin B/L pathway of proteolytic cleavage of viral S protein and by facilitating the interaction between ACE2 and S protein (Ou et al., 2020).

Evidence of HCQ use in COVID-19 from *in vivo* animal models has not been encouraging. Ferrets and hamsters have been found to be permissive to SARS-CoV-2 infection, making good preclinical models. Studies in Syrian hamsters found that HCQ given in a standard dose (6.5 mg/kg) or high dose (50 mg/kg) did not prevent virus transmission and had an insignificant effect on viral replication and disease progression (Kaptein et al., 2020; Rosenke et al., 2020). Nonhuman primates such as rhesus and cynomolgus macaques have been used to develop animal models of COVID-19, resembling human disease, with rhesus macaques developing transient symptomatic disease (Munster et al., 2020; Rockx et al., 2020). Giving high-dose HCQ pre-exposure prophylaxis (30 mg/kg loading dose followed by 15 mg/kg) to cynomolgus macaques did not result in reduction of viral loads (Maisonnasse et al., 2020). Similarly, standard-dose HCQ prophylaxis was found to be ineffective in the rhesus macaque disease model (Rosenke et al., 2020). Thus, the *in vitro* efficacy of HCQ is not replicated *in in vivo* animal models, raising reasonable doubts about its efficacy as prophylaxis for COVID-19.

The dosage of HCQ for adult humans likely to be effective for prophylaxis has been estimated by deriving simulations from pharmacokinetic parameters estimated from healthy individuals and patients with malaria taking HCQ (Al-Kofahi et al., 2020). A regimen of 800 mg loading dose followed by 400 mg twice/thrice weekly for pre-exposure prophylaxis and 800 mg loading dose followed by 600 mg after 6 h and 600 mg daily for 4 days for postexposure prophylaxis has been suggested, which is much higher than required when HCQ is given as prophylaxis against malaria (400 mg once weekly). However, it should be noted that the *in vitro* EC_50_ used for estimations (0.72 µM) in this study was derived from an experiment simulating treatment; EC_50_ for *in vitro* experiments with pretreatment with HCQ, thus simulating pre-exposure prophylaxis, is in a higher range (Liu et al., 2020; Yao et al., 2020).

## Critical Analysis of the Clinical Evidence

Clinical evidence on use of HCQ as chemoprophylaxis (Table 1) is conflicting. HCQ is a widely prescribed drug for rheumatic diseases like systemic lupus erythematosus (SLE) and rheumatoid arthritis, and studies on this group of patients receiving chronic HCQ therapy have provided valuable insights. Initially, due to lack of reports of patients with SLE contracting COVID-19, it was thought that HCQ may have been the reason (Joob and Wiwanitkit, 2020). However, the COVID-19 Global Rheumatology Alliance, a physician-reported registry, has reported more than 600 cases of COVID-19 in patients with rheumatic diseases, including 85 with SLE. In this cohort, 130 patients (51 with SLE) were on long-term antimalarial therapy and use of antimalarials was not associated with protection against hospitalization due to COVID-19 (Gianfrancesco et al., 2020; Konig et al., 2020). In another cohort of 914 patients with 112 chronic HCQ users, HCQ use did not protect against COVID-19 infection (Favalli et al., 2020). Among almost 55,000 patients chronically exposed to antimalarials matched with thrice the number of controls, there was no significant difference in time to COVID-19 hospitalization (Sbidian et al., 2020). Thus, current evidence points that chronic HCQ use does not universally protect patients with rheumatic diseases against COVID-19. However, these results should be interpreted taking into account the limitations of these studies. They are prone to high risk of selection bias with more severe cases more likely to be reported. Other confounders known to affect outcomes such as age, presence of comorbidities, and immunosuppressive treatment may be contributory, for example, in one study, the HCQ arm had a greater proportion of patients who were on corticosteroid therapy (Favalli et al., 2020). Results from these studies cannot be applied to the general population since they only included patients with rheumatic disease.

**TABLE 1:** A summary of published clinical research regarding role of HCQ as prophylaxis against COVID-19.

S. no	Study	Methodology	Results	Limitations
1	(Gianfrancesco et al., 2020)	Cross-sectional case series from a physician-reported registry of patients with rheumatic diseases who have contracted COVID-19; 600 cases from 40 countries	No significant association found between antimalarial therapy and hospitalization after adjusting for sex, age greater than 65 years, rheumatic disease, smoking status, comorbidities, other DMARDs, NSAID use, and glucocorticoid dose	Risk of selection bias due to physician reporting. Risk of bias from unknown confounders. Results cannot be generalized. Cross-sectional analysis, thus patient end points, may have been different in reality
2	(Sbidian et al., 2020)	Retrospective matched cohort study using French national health data; 54,873 cases exposed to antimalarials and 155,689 controls	Hospitalization due to COVID-19 occurred in 128 cases and 195 controls. No significant association of exposure to antimalarial with hospitalization due to COVID-19 on multivariate conditional Cox regression	Retrospective methodology. Risk of bias from unknown confounders cannot be excluded
3	(Favalli et al., 2020)	Survey-based study to ascertain incidence of COVID-19 and its effect on treatment of patients with rheumatic diseases; 914 patients with 112 on chronic HCQ	Incidence of COVID-19 comparable among HCQ and non-HCQ group. Use of biologicals higher in non-HCQ group, and use of corticosteroids higher in HCQ group	Patient-reported data; thus, accuracy cannot be established. Lack of matching between the two groups
4	(Chatterjee et al., 2020)	Case–control study evaluating factors influencing risk of SARS-COV-2 infection in HCW; 378 cases (HCW with COVID-19) and 373 controls	On multivariate analysis, consumption of >/ = 4 maintenance doses of HCQ and use of PPE associated with decreased risk of infection. Dose–response relationship exists between frequency of exposure to HCQ and decrease in risk	Calculated sample size not achieved. Retrospective methodology. No explanation for paradoxical increase in risk with 2–3 doses. Case–controls not matched according to risk of exposure
5	(Bhattacharya et al., 2020)	Retrospective cohort study in 104 HCW (54 on HCQ prophylaxis) who had confirmed contact with a COVID-19–positive case	Distribution of age, sex, degree of exposure, type of exposure, and comorbidities similar in HCQ and non-HCQ groups. HCQ use was associated with 80.7% reduction in risk of acquiring COVID-19 on univariate analysis	Small sample size, retrospective methodology, and confounders not accounted for in univariate analysis
6	(Lee et al., 2020)	Prospective study of outbreak management at a long-term care hospital with HCQ postexposure prophylaxis (400 mg daily) for 14 days	Postexposure prophylaxis completed in >95% without adverse events; 15.6% reported adverse events. All follow-up RT-PCR at the end of 14 days negative	Lack of control group. Index cases were wearing face masks at all times, thus decreasing transmission probability
7	(Gendelman et al., 2020)	Retrospective study on a computerized healthcare database of patients screened for COVID-19	No significant difference between chronic use of HCQ between those positive for COVID-19 (0.23%) vs. those negative for COVID-19 (0.25%)	Retrospective methodology and duration of treatment unknown
8	(Boulware et al., 2020)	Double-blind RCT; 821 asymptomatic participants (719 with confirmed high-risk exposure to COVID-19 contact) randomized to receiving HCQ (414) or placebo (407) within 4 days of exposure for a total of 5 days	Incidence of new COVID-19 did not differ significantly between those taking HCQ (11.8%) vs. placebo (14.3%); 40.1% participants taking HCQ reported side effects; no serious events	Participant-reported data; thus, accuracy cannot be established. Only 18.7% of those labeled to have COVID-19 were had confirmatory RT-PCR
9	(Mitja et al., 2020)	Open-label, cluster randomized trial; 2,314 asymptomatic contacts (exposed within 7 days of enrollment) of 672 index cases randomized to HCQ (1,116) or usual care (1,198)	138 (6.0%) participants had a symptomatic PCR-confirmed COVID-19 episode with no significant difference between the HCQ group (6.2%) and usual care group (5.7%); 51.6% in the HCQ group reported side effects with no serious events	Blinding not performed; 12.2% participants had a positive baseline RT-PCR
10	(Rajasingham et al., 2020)	Double-blind RCT; 1483 HCW with ongoing COVID-19 exposure randomized 2:2:1:1 to receiving once/twice weekly HCQ or placebo for 12 weeks	97 (6.5%) participants developed COVID-19. Incidence of COVID-19 was 0.27, 0.28, and 0.38 events per person year in once-weekly HCQ, twice-weekly HCQ, and placebo group, respectively (no significant difference). Median HCQ blood concentrations did not differ among COVID and non-COVID cases. One serious AE (SVT) in the twice-weekly HCQ group	Participant-reported data; thus, accuracy cannot be established. Only 18% of those labeled to have COVID-19 had a confirmatory positive RT-PCR; 39% had negative RT-PCR during illness
11	(Abella et al., 2020) (PATCH trial)	Double-blind RCT; 132 HCW randomized to receive HCQ (600 mg daily) or placebo for 8 weeks; 125 evaluated for primary outcome	No significant difference in infection rate among participants receiving HCQ (6.3%) vs. placebo (6.6%). 45% in the HCQ group had mild side effects. Median change in QTc interval was not significantly different in both groups	Small sample size, trial terminated early. Study population comprised young HCW, thus results not generalizable
12	(Garcia-Albeniz et al., 2020)	Meta-analysis of three randomized trials on HCQ prophylaxis	Pooled risk ratio estimate with use of HCQ as prophylaxis was 0.78 (95% CI: 0.61–0.99)	End point of PCR-confirmed disease pooled with different end point of clinical disease. Results of trials with different methodologies pooled together

On the other hand, few observational studies report a positive preventive effect of HCQ. In a case–control study from India involving HCW involved in care of COVID-19 patients, consumption of four or more maintenance doses of HCQ (400 mg once weekly) for prophylaxis was associated with lower risk of contracting COVID-19 after adjusting for sex, use of PPE, performance of endotracheal intubation, and COVID-19 testing date (Chatterjee et al., 2020). However, the dose–response curve noted a paradoxical increase in risk of infection after two to three doses, which cannot be well explained and casts doubts on the actual presence of a protective effect. Another retrospective cohort study in HCW who had confirmed contact with a COVID-19 case reported HCW who took HCQ were at a lower risk of infection; however, this was only observed on univariate analysis (Bhattacharya et al., 2020). Both these studies were prone to bias due to their retrospective methodology, small sample size, and presence of confounders, such as duration/degree of exposure to COVID-19 patients. In a study from South Korea, HCQ was administered as postexposure prophylaxis to 211 individuals in a long-term care hospital after exposure to a confirmed COVID-19 case. None developed COVID-19, and acceptability was good, although this apparent protective benefit cannot be confirmed due to lack of a control group and use of facemask by the index case (Lee et al., 2020).

Several randomized trials evaluating HCQ prophylaxis in HCW are currently ongoing (Agarwal et al., 2020), with the results of a few of them now available (Table 1). The first trial, published in June, investigated HCQ as postexposure prophylaxis in adult HCW with high/moderate-risk exposure to a confirmed case of COVID-19. Participants were randomized to HCQ or placebo within 4 days of exposure with the dosing regimen adapted from a pharmacokinetic simulation study. The incidence of new COVID-19 did not differ between those receiving HCQ and placebo. Another trial with a similar design, investigating pre-exposure prophylaxis in HCW with once weekly or twice weekly HCQ, reported no protective benefit compared to placebo (Rajasingham et al., 2020). Both these trials had a pragmatic design, due to which certain limitations made it difficult to draw definite conclusions—only a few of the trial participants had an RT-PCR confirming their COVID-19 diagnoses, and the rest were labeled based on a symptom-based definition. Due to the trial population comprising mostly young HCW, the incidence of COVID-19 may have been underestimated due to asymptomatic infections being missed. However, other trials have also reported a similar result. A cluster randomized trial from Spain reported no benefit of HCQ postexposure prophylaxis (Mitja et al., 2020), while randomizing HCW to HCQ pre-exposure prophylaxis (600 mg daily) or placebo also did not result in any protective benefit from COVID-19 (Abella et al., 2020). Side effects were encountered in 40–50% participants taking HCQ; however, these were most commonly mild gastrointestinal adverse events like nausea, loose stools, and abdominal discomfort. One serious adverse event of syncope and supraventricular tachycardia was reported (Rajasingham et al., 2020). A recent meta-analysis pooled the results of three of these clinical trials and reported a significant risk reduction of 20% with HCQ use (Garcia-Albeniz et al., 2020), but there were glaring inaccuracies in the analysis as data with different end points (PCR-confirmed disease v/s clinically compatible disease) and trials with different methodologies, for example, results of pre-exposure and postexposure prophylaxis trials were pooled. Thus, the conclusions may not be reliable. In summary, the results from these trials indicate that HCQ in its current dosage does not seem to provide a prophylactic benefit against COVID-19.

## Discussion

### The Conundrum of Dosing for Hydroxychloroquine Prophylaxis

One reason that may explain why the apparent *in vitro* efficacy of HCQ could not be replicated in preclinical *in vivo* studies and in clinical trials is a *pharmacokinetic* failure. 4-aminoquinolones have peculiar pharmacokinetic properties, which make it difficult to accurately estimate pharmacological parameters. HCQ is well observed orally (74% bioavailability) and has an overwhelmingly large volume of distribution, indicating extensive sequestration into tissues (Tett et al., 1988; Tett et al., 1989). Due to this reason, the volume of distribution dictates its pharmacokinetics, leading to a long half-life (∼44 days), despite good clearance. Further, around 45% of HCQ in plasma is bound to plasma proteins, with >90% bound to albumin (Tett et al., 1988). It has been seen that measured plasma drug concentrations of HCQ are much more variable than measured whole blood concentrations (Gustafsson et al., 1983; Tett et al., 1988; Blanchet et al., 2020), probably due to the release of the drug from WBCs and platelets during sample processing. This has been observed even with modifications in the separation procedure like increasing centrifugation speed and decreasing time to separation. Thus, whole blood HCQ levels are a more accurate parameter for pharmacokinetic estimations.

For clinical efficacy, appropriate concentrations of the drug should be achieved at its target site. Current knowledge suggests that since HCQ blocks viral entry, its site of action would be extracellular lung tissue and intracellularly in type 1 pneumocytes. The *in vitro* EC_50_ for HCQ prophylaxis lies in the range of 4.51–5.85 µM (Liu et al., 2020; Yao et al., 2020), while the *in vivo* EC_50_ is unknown. It is important to note that this EC_50_ value has been measured in the extracellular cell culture media. Since HCQ is concentrated within tissues in the acidic intracellular compartment such as the lysosomes, endosomes, and golgi apparatus, the above EC_50_ values should be extrapolated to free extracellular tissue concentrations, which would be in turn in equilibrium with the free (unbound) plasma concentration. Correlating these values with whole lung HCQ concentrations (Yao et al., 2020) is likely to be inaccurate since they would include the high intracellular concentration (Fan et al., 2020). For example, in the case of SLE and other rheumatic diseases, HCQ is given at a maximum dose of 400 mg/day, which maintains whole blood levels in the range of 648–917 ng/ml (1.93–2.73 µM) (Blanchet et al., 2020; Mathian et al., 2020). With a blood-to-plasma ratio of HCQ concentration being 7.2 and close to 50% of plasma HCQ being protein bound (Tett et al., 1988), the free plasma HCQ concentration (which would be in equilibrium with the free extracellular tissue concentration) comes out to be in the range of 45–64 ng/ml (0.13–0.19 µM), which is considerably lower than the *in vitro* EC_50_. This may explain why patients with rheumatological diseases on chronic HCQ do not get a protective benefit.

Currently used dosing regimens for HCQ prophylaxis (Al-Kofahi et al., 2020) have been estimated from a population pharmacokinetic model based on plasma HCQ levels in patients with malaria and healthy volunteers (Lim et al., 2009). This estimation can lead to inaccuracies due to multiple reasons. As malaria is a bloodstream infection, the site of action of HCQ is in the blood compartment itself; but the site of action in COVID-19 is within the lung tissue. Further, the EC_50_ values used in this estimation have been derived from a treatment experiment. Indeed, whole blood levels of HCQ in human participants receiving the suggested regimen for pre-exposure prophylaxis (800 mg followed by 400 mg biweekly) were only 200 ng/ml (0.59 µM), corresponding to a free plasma concentration of 13.9 ng/ml (0.04 µM) much smaller than EC_50_ values (Rajasingham et al., 2020). Moreover, a recently published population pharmacokinetic model derived from whole blood concentrations of HCQ in treated COVID-19 patients found that current dosing regimens were inadequate for a corresponding *in vitro* EC_50_ of 4.51 µM, and body weight was a significant factor influencing HCQ clearance (Thémans et al., 2020). According to this model, much higher doses would be required for a clinical effect.

Thus, we currently do not have accurate predictions of the dosage of HCQ required for chemoprophylaxis. Measurement of HCQ blood levels and, if feasible, lung fluid levels (e.g., through bronchoalveolar lavage specimens) in participants of clinical trials of HCQ prophylaxis combined with the use of comprehensive pharmacokinetic models may provide us with answers. However, there is a flip side to this coin. Higher doses of HCQ may bring with them the risk of serious toxicity, especially cardiotoxicity in the form of QT_C_ prolongation and cardiac arrhythmias. Extrapolating risk of QT_C_ prolongation with HCQ from CQ models in children with malaria led to the conclusion that HCQ doses in the range of >/ = 800 mg BID may have significant risk (Garcia-Cremades et al., 2020). Currently used doses of HCQ are usually safe in outpatient settings (Lofgren et al., 2020), but use of HCQ to treat COVID-19 patients in inpatient and ICU settings has led to safety concerns (Bonow et al., 2020; Jeevaratnam, 2020). Many such patients are elderly and may have pre-existing cardiovascular disease. COVID-19 leads to viral myocardial injury in 7–23% patients (Pirzada et al., 2020); further patients are commonly treated with concomitant QT_C_-prolonging drugs like azithromycin; this increases the risk of cardiotoxic events (Agarwal et al., 2020; Padilla et al., 2020). Indeed, observational studies have reported that HCQ for treatment of COVID-19 leads to critical QT_C_ prolongation (a marker for risk of Torsades de pointes) in 20–36% cases, frequently requiring drug discontinuation to avoid fatal arrhythmias (Bessière et al., 2020; Chorin et al., 2020; Mercuro et al., 2020). A recent meta-analysis noted an increased risk of cardiac arrhythmias with HCQ use compared to standard of care (Elavarasi et al., 2020). Moreover, instances of ventricular arrhythmias have occurred due to HCQ as reported by observational cohorts (Chorin et al., 2020; Mercuro et al., 2020) and pharmacovigilance data (Pharmacovigilance Memorandum. Reference ID: 4610984, 2020). Besides cardiovascular adverse effects, there have been sporadic cases of other serious adverse events including neuropsychiatric events, hepatitis, cytopenias, rhabdomyolysis, and acute kidney failure that have been attributed to HCQ (Garcia et al., 2020; Pharmacovigilance Memorandum. Reference ID: 4610984, 2020). It must be noted that most of these observations are from HCQ used as a therapy for COVID-19, but they may have implications for chemoprophylaxis as well owing to the doubtful efficacy and long half-life of HCQ (Agarwal et al., 2020). Therefore, it becomes important to balance risk with possible benefit.

Another theory that has been suggested cautioning against use of CQ/HCQ is that of *hormesis*, that is, a biphasic effect on viral replication with stimulation at lower doses and inhibition at higher doses (Calabrese et al., 2021). A paradoxical increase in viral load may occur due to a hormetic effect due to preconditioning (acquired resilience) of viral particles after exposure to certain doses of CQ/HCQ (Calabrese, 2016). This is, however, based on observations of the effect of CQ on neuroprotection and SARS-CoV-1 viral growth (Keyaerts et al., 2004); currently, there is no evidence regarding a hormetic effect of CQ/HCQ on SARS-CoV-2.

### Other Caveats in Knowledge: A Role of TMPRSS2?

Several aspects of the role of HCQ in COVID-19 prophylaxis are currently incompletely understood. The translation of *in vitro* activity to *in vivo* activity is a complex process affected by a multitude of factors. As discussed above, the dosing of HCQ is likely contributory. However, other factors may also be playing a role. A recent study evaluating the *in vivo* effect of HCQ in Syrian hamster model of COVID-19 reported that HCQ did not reduce viral loads or affect viral transmission even when given at a high dose of 50 mg/kg/day (Kaptein et al., 2020). Lung tissue concentration of HCQ was derived from a mean trough plasma HCQ concentration using previously known estimates, and both cytosolic and interstitial lung tissue concentrations were found to be 5.4 µM (Kaptein et al., 2020), which is in line with the *in vitro* EC_50_; thus, tissue concentrations were not a limiting factor. An explanation for this discrepancy is interference by TMPRSS2. Airway epithelium lacks sufficient expression of cathepsin B/L; thus, the main mechanism of SARS-CoV-2 entry is via S protein activation by TMPRSS2. HCQ has no effect on the action of TMPRSS2, and it has been seen that the inhibitory effect of HCQ on viral entry is effectively attenuated by TMPRSS2 expression (Ou et al., 2020). This also gives an explanation as to why HCQ failed to demonstrate efficacy on a reconstituted airway epithelium model (Maisonnasse et al., 2020); airway epithelium, unlike Vero cells, expresses high amounts of TMPRSS2. Further, it was seen that ablation of the furin *preactivation* site on the S protein reduced the dependence on TMPRSS2 (Ou et al., 2020). Thus, using inhibitors of TMPRSS2 and furin along with HCQ can theoretically inhibit SARS-CoV-2 entry in a comprehensive manner and will likely have additive protective benefit. TMPRSS2 is especially an attractive target since it is not required for normal homeostasis (Ts et al., 2006); its inhibitor—camostat mesilate—is approved for human use in Japan for chronic pancreatitis, and camostat has been demonstrated to protect mice models from SARS-CoV infection (Zhou et al., 2015).

### Are There Alternatives to Chemoprophylaxis?

Currently, there is no direct evidence to suggest alternative drugs for chemoprophylaxis in COVID-19. Theoretically, other molecules inhibiting SARS-CoV-2 entry may be effective agents. As discussed above, the TMPRSS2 inhibitor camostat mesilate and furin inhibitors may be worth looking into. Further, molecular docking studies have identified that the S protein of SARS-CoV-2 may bind to additional molecules like heat shock protein A5 (HSPA5/GRP78) (Ibrahim et al., 2020), and certain natural compounds like phytoestrogens may inhibit this interaction (Elfiky, 2020). However, it must be reiterated that use of these agents is chemoprophylaxis is currently only a hypothesis.

## Conclusion

Certain aspects of HCQ show promise for a chemoprophylactic agent against COVID-19; it has a plausible mechanism of action inhibiting viral entry, and it is cheap and widely available. However, current evidence, including preclinical animal models and clinical trials, suggest that HCQ in its current form is not effective for COVID-19 chemoprophylaxis. Certain questions however remain:

- Should guidelines recommending HCQ prophylaxis be revised?

During the early months of the pandemic, recommending HCQ for prophylaxis based on preclinical evidence may be justified due to the nature of the circumstances. But guidelines need to be updated in light of new evidence. Since clinical trials have shown that HCQ prophylaxis is not showing clinical benefit, current guidelines need to be revised accordingly.

- Should HCQ be tried in clinical trials using a different dosing regimen with higher doses?

In its traditional doses, HCQ has largely been a safe drug. Higher doses however have the potential to cause serious adverse events including cardiac events such as QT_C_ prolongation, ventricular arrhythmias, and noncardiac events. Pharmacovigilance has already detected in sporadic cases of serious adverse events with HCQ prophylaxis (National Task Force for COVID-19, 2020; Pharmacovigilance Memorandum. Reference ID: 4610984, 2020). Thus, in the setting of questionable efficacy, trying a higher dose of HCQ in clinical trials cannot be justified.

 In conclusion, based on currently available research, looking beyond HCQ for COVID-19 chemoprophylaxis may prove to be a better path.

## Author Contributions

Authors PR and UB conceptualized the study. Authors MA and AM conducted the review of literature. Author MA prepared the primary draft of the manuscript and prepared the figure and table. Authors PR, UB, AM, and MA performed critical revision of the primary manuscript draft for intellectual content. Author PR supervised the study.

## Conflict of Interest

The authors declare that the research was conducted in the absence of any commercial or financial relationships that could be construed as a potential conflict of interest.

## Abbreviations

HCQ, hydroxychloroquine; CQ, chloroquine; SARS-CoV-2, severe acute respiratory syndrome coronavirus 2; MERS-CoV, Middle Eastern respiratory syndrome coronavirus; PPE, personal protective equipment; HCW, healthcare workers; G6PD, glucose-6-phosphate dehydrogenase; IL, interleukin; TNF, tumor necrosis factor; TLR, toll-like receptor; USFDA, U.S. Food and Drug Administration.
